# CRTAM enhances T cell antimicrobial activity, particularly CD8^+^ T cells, against *Mycobacterium tuberculosis* infection

**DOI:** 10.3389/fimmu.2026.1964260

**Published:** 2026-10-23

**Authors:** Ying Peng, Juechu Wang, Xuejiao Ji, Guixian Huang, Liying Zhu, Yuan Wu, Xiaocui Wu, Wei Sha, Feifei Wang, Hongbo Shen

**Affiliations:** 1 Shanghai Clinical Research Center for Infectious Disease (tuberculosis), Shanghai Key Laboratory of Tuberculosis, Shanghai Pulmonary Hospital, Institute for Advanced Study, Tongji University School of Medicine, Shanghai, China; 2 Key Laboratory of Medical Molecular Virology (MOE/NHC/CAMS), Shanghai Institute of Infectious Disease and Biosecurity, Department of Medical Microbiology and Parasitology, School of Basic Medical Sciences, Shanghai Medical College, Fudan University, Shanghai, China; 3 Department of Clinical Laboratory, Shanghai Pulmonary Hospital, Tongji University School of Medicine, Shanghai, China; 4 Shanghai Sci-Tech Inno Center for Infection and Immunity, Shanghai, China

**Keywords:** CD8^+^ T cells, CRTAM, *Mycobacterium tuberculosis*, protective immune molecule, T cells

## Abstract

**Background:**

The mechanisms underpinning protective host immunity against *Mycobacterium tuberculosis* (*Mtb*) are not fully elucidated. Resisters, individuals with prolonged exposure to *Mtb* who remain negative on repeated immunological testing for *Mtb* infection, provide a unique opportunity to investigate protective immune responses. This study systematically investigated the functional characteristics of CXCR3^+^CD107a^+^ effector cells in Resisters to identify molecules potentially associated with protective anti-mycobacterial immunity.

**Methods:**

mRNA sequencing was employed to investigate the transcriptional characteristics of CXCR3^+^CD107a^+^ effector lymphocytes from Resisters and tuberculosis patients. Flow cytometry was utilized to assess CRTAM expression and effector phenotypes, and colony-forming units assays quantified the bacterial burdens in infected macrophages. *Crtam*-deficient and wild-type mice were used to investigate the function of CRTAM to host control of mycobacterial infection *in vivo*.

**Results:**

mRNA sequencing of CXCR3^+^CD107a^+^ effector lymphocytes isolated from Resisters revealed elevated CRTAM expression, which positively correlated with anti-mycobacterial effector molecules. CRTAM levels were lower in patients with chronic *Mtb* infection. Mycobacterial stimulation rapidly increased CRTAM expression, particularly in CD8^+^ T cells. CRTAM expression and agonistic CRTAM stimulation were associated with enhanced T-cell effector responses and inhibition of intracellular mycobacterial growth. *Crtam*-deficient mice showed increased susceptibility to mycobacterial infection, whereas recombinant CRTAM administration conferred significant immune protection.

**Conclusions:**

These findings identify CRTAM as a protective immune-associated molecule that contributes to anti-mycobacterial effector responses. The recombinant CRTAM findings provided initial *in vivo* proof-of-concept that CRTAM can modulate infection-associated outcomes during *Mtb* infection, supporting its further investigation as an immune-regulatory molecule.

## Introduction

Tuberculosis (TB), caused by *Mycobacterium tuberculosis* (*Mtb*) and its closely related complex, remains one of the deadliest infectious diseases globally (1). According to the WHO Global Tuberculosis Report 2025 (2), TB resulted in over 1.2 million deaths and approximately 10.7 million new cases in 2024, maintaining its status as the leading cause of death from a single infectious agent. Thus, TB presents a significant global public health challenge, emphasizing the need to elucidate effective protective immune responses against *Mtb* infection (3, 4).

Recent studies have focused on a unique group of individuals known as “Resisters,” such as household contacts of TB patients, who are highly exposed to *Mtb* yet test negative for both IFN-γ release assays and tuberculin skin tests (5–8). These Resisters exhibit IgM and class-switched IgG antibody responses, along with non-IFN-γ T cell responses to *Mtb*-specific antigens, including ESAT-6 and CFP-10, providing immunological proof of *Mtb* exposure (5, 9). Unlike those with classical latent TB infection (LTBI), Resisters display enhanced antibody-dependent activity and distinct *Mtb*-specific IgG Fc profiles, suggesting a robust innate or innate-like immune system capable of rapidly clearing the infecting bacteria without provoking an adaptive T-cell response. This underscores the significance of Resisters in studying protective immune responses against TB (10). Investigating the immunological features of Resisters may reveal key immune factors providing protection against *Mtb* infection.

Effector cells capable of rapid chemotactic migration into the lungs are essential for clearing early-stage *Mtb* infection (11). Studies have confirmed that upon *Mtb* infection, a significant number of CXCR3-expressing cells are rapidly recruited to the lungs (12, 13). This chemokine receptor, which primarily responds to interferon-induced ligands, mediates the precise homing of Th1 cells to infection sites (14, 15). Among the critical effector cells eliminating *Mtb* are cytotoxic lymphocytes. CD107a, indicating degranulation, is a key marker for NK and CD8^+^ T cell cytotoxic activity (16, 17). Our previous studies demonstrated that CXCR3^+^CD107a^+^ cells express activation-related molecules, such as CD137, involved in regulating effector functions (18).

To gain insight into the cellular mechanisms underlying effective host immunity against *Mtb*, we examined the transcriptional profiles of CXCR3^+^CD107a^+^ effector cells in Resisters, a population characterized by effective control of *Mtb* infection. We sought to identify molecular features of these effector cells that may contribute to protective anti-tuberculosis immunity. This analysis identified CRTAM (Class I-restricted T cell-associated molecule, also known as CD355), a cell-surface molecule implicated in T cell activation and cytotoxicity, as a candidate regulator of effector T cell function. These findings prompted us to investigate the contribution of CRTAM to T cell-mediated anti-tuberculosis immunity and its potential relevance to host-directed TB immunotherapy.

## Methods

### Human subjects

Whole blood samples were collected from patients with active pulmonary TB (ATB), which recruited at Shanghai Pulmonary Hospital of Tongji University (Shanghai, China). Uninfected volunteers without clinical and immunological evidence of ATB or Resisters were recruited as healthy control (HC) (**Table 1**). Resisters in this study were defined as individuals who had been in close contact with patients with pulmonary tuberculosis for long times and remained negative on at least two repeated interferon- gamma release assay (IGRA) over a minimum follow-up period of 6 months. This definition was based on the concept of the Resister phenotype described previously (7, 8). All participants were tested for human immunodeficiency virus (HIV), hepatitis C virus (HCV), and hepatitis B virus (HBV). Individuals with HIV, HCV, and HBV infection and other infectious diseases or cancers were excluded.

**Table 1:** Demographic and clinical characteristics of human subjects.

Characteristics	Healthy controls (HC)	Tuberculosis patients (TB)	Resisters
Number of subjects	104	42	3
Age (mean ± SEM)	38.85 ± 2.04	41.10 ± 1.40	37.33 ± 2.96
Gender (M/F)	62/42	27/15	3/0
Chest X-ray positive	0/104	42/42	0/3
Sputum culture positive	0/104	42/42	0/3
IGRA^a^ positive	0/104	42/42	0/3
Close contact with tuberculosis patients (≥5 years)	0/104	0/42	3/3
Follow-up duration (months)	N/A^b^	N/A	6-12
Repeated IGRA testing	N/A	N/A	2-3

^a^interferon-γ release assay; ^b^N/A, not applicable.

### Peripheral blood mononuclear cells (PBMCs) isolation and cell culture

PBMCs were isolated from EDTA-treated blood using Ficoll-Paque plus density gradient centrifugation and cultured in RPMI-1640 media supplemented with 10% fetal bovine serum (FBS, Gibco, USA) and 100 U/mL of penicillin-streptomycin in a 5% CO_2_ incubator at 37°C, as described in our previous studies (19).

PBMCs were resuspended in RPMI-1640 supplemented with 10 ng/mL M-CSF (PeproTech, USA). After 6–7 days at 37 °C, 5% CO_2_, with a half-medium change on day 3-4, non-adherent cells were removed by gentle washing. The firmly adherent, enlarged cells were used as human monocyte-derived macrophages (hMDMs).

The THP-1 cell line was obtained from the Cell Bank of the Chinese Academy of Sciences (Shanghai, China). THP-1 cells were cultured in RPMI-1640 media. THP-1 cells were treated with 50 ng/mL phorbol 12-myristate 13-acetate (PMA, Sigma-Aldrich) for 24 h to induce differentiation into macrophages, then washed three times with pre-warmed PBS and maintained in medium at 37 °C for further use. All of the cell culture media contained 10% FBS and 100 U/mL of penicillin-streptomycin. Cells were cultured in a 5% CO_2_ incubator at 37°C.

### Co-culture system of effector cells and target cells *in vitro*

Following overnight incubation of PBMCs in pre-coated (anti-CD3/CD28 antibodies, 2 μg/mL, Invitrogen) 96-well U-bottom plates, CRTAM^+^CD3^+^ T cells were enriched using MACS Separation columns (Miltenyi Biotech, Germany) according to the manufacturer’s protocol, then used as effector cells.

PBMCs or purified CRTAM^+^CD3^+^ T cells (1-5×10^5^ cells/well) were co-cultured with THP-1or hMDMs target cells (1-5×10^4^ cells/well) at an effector-to-target (E:T) ratio of 10:1 in 96-well plates. Negative counterparts of CRTAM^-^CD3^+^ T cells were included in these co-culture systems and served as negative controls. For functional assays, cells were treated with 10 μg/mL anti-human CRTAM mAb (Cr24.1, BioLegend), 10 μg/mL mouse IgG2a isotype control (MG2a-53, BioLegend), or 20 μg/mL recombinant human CRTAM protein (MCE, USA). At the indicated time points, cells were harvested for flow cytometry analysis or Colony-Forming Units (CFU) assays.

### Lentiviral transduction

Cells were transduced with lentiviral particles encoding CRTAM or shRNA targeting CRTAM (Tsingke Biotech, China) at a multiplicity of infection (MOI) of 10 in the presence of 8 µg/mL polybrene (Yeasen, China). For lentiviral transduction of sorted CD3^+^ T cells, plates were pre-coated with 2 μg/mL anti-CD3/CD28 antibodies (Invitrogen, USA) for 24 h prior to transduction. CRTAM-overexpressing or CRTAM-silencing lentivirus was then added to the cells at an MOI of 10. After 24 h, the medium was replaced with fresh medium supplemented with IL-2 (10 ng/mL, R&D). The cells were then harvested three days later. Transduction efficiency and CRTAM expression were confirmed by quantitative PCR.

### Mycobacteria culture, infection and CFU assay

*Mycobacterium tuberculosis* H37Rv (ATCC 27294) and *Mycobacterium bovis* BCG (ATCC 35733) were grown in Difco Middlebrook 7H9 broth supplemented with 10% OADC (BD, USA), 0.05% (v/v) Tween 80 and 0.2% (v/v) glycerol in a 5% CO_2_ incubator at 37°C. *Mycobacterium smegmatis* (*M. smegmatis*) (ATCC 607) were grown in LB medium at 37°C.

THP-1-derived macrophages and hMDMs were infected with H37Rv at an MOI of 1 for ~4 h, BCG at an MOI of 10 for ~4 h, or *M. smegmatis* at an MOI of 1 for ~2 h. After infection, extracellular bacilli were removed by washing three times with pre-warmed PBS, and the infected cells were cultured for 3 days or given time. Then, the infected cells were lysed in sterile PBS with sodium dodecyl sulfate (SDS, Sigma-Aldrich) in working concentration of 0.3 g/L. Serial dilutions were performed for quantitative culture. Mycobacteria viability was quantified via counting CFU as we previously described (20).

Slow-growing H37Rv or BCG reached logarithmic phase in a 2- to 4-week culture, and *M. smegmatis* within 2–4 days. Cells or cultures were measured for mycobacterial colony counts in the biosafety level (BSL)-II+ facility (BSL3-equivalent lab for handling aerosol-free samples from patients) in Shanghai Pulmonary Hospital of Tongji University.

### Flow cytometry

Flow cytometry was performed as previously described (21). Briefly, cells were harvested and stained with Zombie Aqua™ Fixable Viability Kit (BioLegend, USA) to assess viability. Cell surface staining was performed using the following monoclonal antibodies: anti-human CD3-PerCP-Cy5.5 (SK7), anti-human CD8-AF700 (RPA-T8), anti-human CD4-BV510 (OKT4), anti-human CXCR3-APC-Cy7 (G025H7), anti-human CRTAM-PE (Cr24.1), anti-human CD27-APC (O323), anti-human CD45RA-BV785 (H100), anti-mouse CD3-PerCP-Cy5.5 (145-2C11), anti-mouse CD8-AF700 (53-6.7), anti-mouse CD69-BV605 (H1.2F3), and anti-mouse CRTAM-PE (11-5/CRTAM), all from BioLegend. After two washes, cells were fixed and permeabilized for intracellular cytokine staining with anti-human Granzyme B-FITC/PB (QA16A02), anti-human IFN-γ-BV785 (4SB3), anti-human Perforin-PerCP-Cy5.5/PE-Cy7 (G9), anti-human granulysin-APC (DH2), anti-human CD107a-PerCP-Cy5.5 (H4A3) from BioLegend, and anti-human IL-17A-PE (12-7178-42; Invitrogen). Data were acquired on a Beckman Coulter DxFLEX flow cytometer and analyzed using FlowJo and CytExpert software.

### RNA isolation and real-time quantitative PCR

Total RNA was extracted from cultured cells using the Quick-RNA Isolation Kits (Zymo Research, USA) according to the manufacturer’s instructions. For mRNAs, First-strand cDNA was synthesized with PrimeScript™ RT reagent Kit with gDNA Eraser (TaKaRa, Japan) and analyzed by real-time quantitative PCR with SYBR Premix Ex TaqTM (TaKaRa, Japan) following the manufacturer’s protocols. Relative expression of target gene was normalized to that of the house-keeping gene GAPDH. The primer sequences used for quantitative real-time PCR were listed below and synthesized from Tsingke Biotech: CRTAM-F: 5’-GGCCAGACGCTCACTCTAAA-3’, CRTAM-R: 5’-GCAGGGTTACGTTAGGCACA-3’; GAPDH-F: 5’-AGGTCGGTGTGAACGGATTTG-3’, GAPDH-R: 5’-TGTAGACCATGTAGTTGAGGTCA-3’. Fold change was calculated with the 2-^ΔΔCT^ method.

### RNA-seq methods

Two RNA^-^seq datasets were analyzed in this study. The first dataset included CXCR3^+^CD107a^+^ double^-^positive (DP) cells and CXCR3^+^CD107a^-^ single^-^positive (SP) cells from 3 Resister individuals and 4 individuals with ATB. The second dataset included CD3^+^CRTAM^+^ and CD3^+^CRTAM^-^ T cells from 5 independent donors, with both populations obtained from each donor.

Following quality control and filtering, sequencing reads were aligned to the human reference genome (hg19) using HISAT2. Alignment and sequencing quality were assessed using RSeQC. Gene expression data were analyzed using DESeq2, including normalization and differential expression analysis. Differentially expressed genes were defined as those with *p < 0.05* and |log2 fold change| > 1. Genes with log2 fold change > 1 were considered upregulated and those with log2 fold change < -1 were considered downregulated. *p* values were adjusted for multiple testing using the Benjamini-Hochberg (BH) method, and adjusted *p* values (padj) were reported.

GO and KEGG enrichment analyses were performed to characterize the biological functions and signaling pathways associated with differentially expressed genes. GO enrichment analysis was performed for the biological process (BP), molecular function (MF), and cellular component (CC) categories. Enrichment results with *p < 0.05* were considered statistically significant.

### Mouse infection

All the mice used in this study were 6-8-week-old female C57BL/6 mice. The *Crtam^−/−^*mice were on a C57BL/6 genetic background and were obtained from GemPharmatech (China). Wild-type (WT) mice were used as controls. WT mice were not littermate controls. Mice were housed in a BSL-3 facility (12-h light/dark cycle, 50 ± 5% humidity, 25-27 °C).

For infection experiments, mice were infected with 1 × 10^4^ *M. tuberculosis* H37Rv, 1 × 10^6^ *M. bovis* BCG or 1 × 10^6^ *M. smegmatis*. Four weeks (H37Rv, BCG group) or 3 days (*M. smegmatis* group) post-infection, animals were sacrificed for bacterial CFU assays, histopathology, and tissue immunofluorescence.

For treatment experiment, wild-type mice were intravenously challenged with 1 × 10^4^ CFU *M. tuberculosis* H37Rv. The mice then received intraperitoneal injections of recombinant mouse CRTAM protein (50 μg in 200 μL per mouse, MCE, USA) or PBS every two days, for a total of six administrations. The first dose was administered two days prior to H37Rv challenge. Animals were sacrificed for bacterial CFU assays and histopathology two days after the final dose.

Animals were randomly assigned to the experimental groups, each animal represented an independent biological replicate. No predefined exclusion criteria were applied, and no animals were excluded from the analysis. Investigators were blinded to group allocation during CFU enumeration and histopathological quantification.

Tissue sections from *Mtb*-infected or control mice were fixed by inflating the tissues with 4% formaldehyde, sectioned and stained with hematoxylin-eosin (H&E), or Ziehl-Neelsen staining. Slides containing histological sections were scanned with Leica biosystems. Histopathological changes were assessed in a blinded fashion using a predefined semiquantitative scoring system (0-4), based on leukocyte infiltration, hemorrhage, alveolar wall thickening, and alveolar edema. A score of 0 indicated no apparent pathological changes, whereas scores of 1, 2, 3, and 4 represented mild, moderate, severe, and very severe pulmonary lesions, respectively.

### Statistical analysis

Statistical analysis was performed by using GraphPad Prism 9.0 software (GraphPad Software, CA, USA). The distribution of the data was assessed using the Shapiro-Wilk test before applying parametric tests, where applicable. For comparisons between two groups, two-tailed paired or unpaired Student’s *t*-tests were used as appropriate, with paired analyses applied to measurements obtained from the same donor or experimental subject. For independent-group comparisons, homogeneity of variance was assessed where relevant. One-way ANOVA was used for comparisons involving multiple groups with a single independent variable. For experiments involving two factors, two-way ANOVA or a mixed-effects model was used according to the experimental design, with repeated measures accounted for when measurements were obtained from the same donor or experimental subject. Appropriate *post hoc* multiple-comparisons tests, including Šídák’s or Dunnett’s tests, were applied according to the experimental design. When the assumptions of parametric tests were not met, appropriate non-parametric tests were used, including the Mann-Whitney *U* test, Wilcoxon matched-pairs test, or Kruskal-Wallis test with Dunn’s multiple-comparisons test. *p < 0.05* was considered to indicate a statistically significant difference.

## Results

### CRTAM highly expressed in CXCR3^+^CD107a^+^ effector lymphocytes of resisters under *Mtb* tuberculin administration and co-expressed with effector cytokines

To identify molecular components associated with host-protective immune response against *Mtb* infection, we transcriptionally profiled CXCR3^+^CD107a^+^ effector lymphocytes isolated from Resisters and ATB individuals after *Mtb* tuberculin administration during skin test (18) (**Figure 1A**). We found that CRTAM expression levels in CXCR3^+^CD107a^+^ (double-positive, DP) cells were ~3-fold of those in CXCR3^-^CD107a^-^ (double-negative, DN) counterparts isolated from the same Resister donors (**Figures 1B, C**). However, there were no significant differences in CRTAM expression levels between CXCR3^+^CD107a^+^ cells and their counterparts of CXCR3^-^CD107a^-^ isolated from ATB patients (**Figure 1C**). Moreover, the average expression level of CRTAM in CXCR3^+^CD107a^+^ cells in ATB patients was about 25% of that in Resisters (**Figure 1C**). These results implied that CRTAM might be associated with protective immune response of Resisters against *Mtb* infection, and that its expression was suppressed during chronic *Mtb* infection.

**Figure 1:**
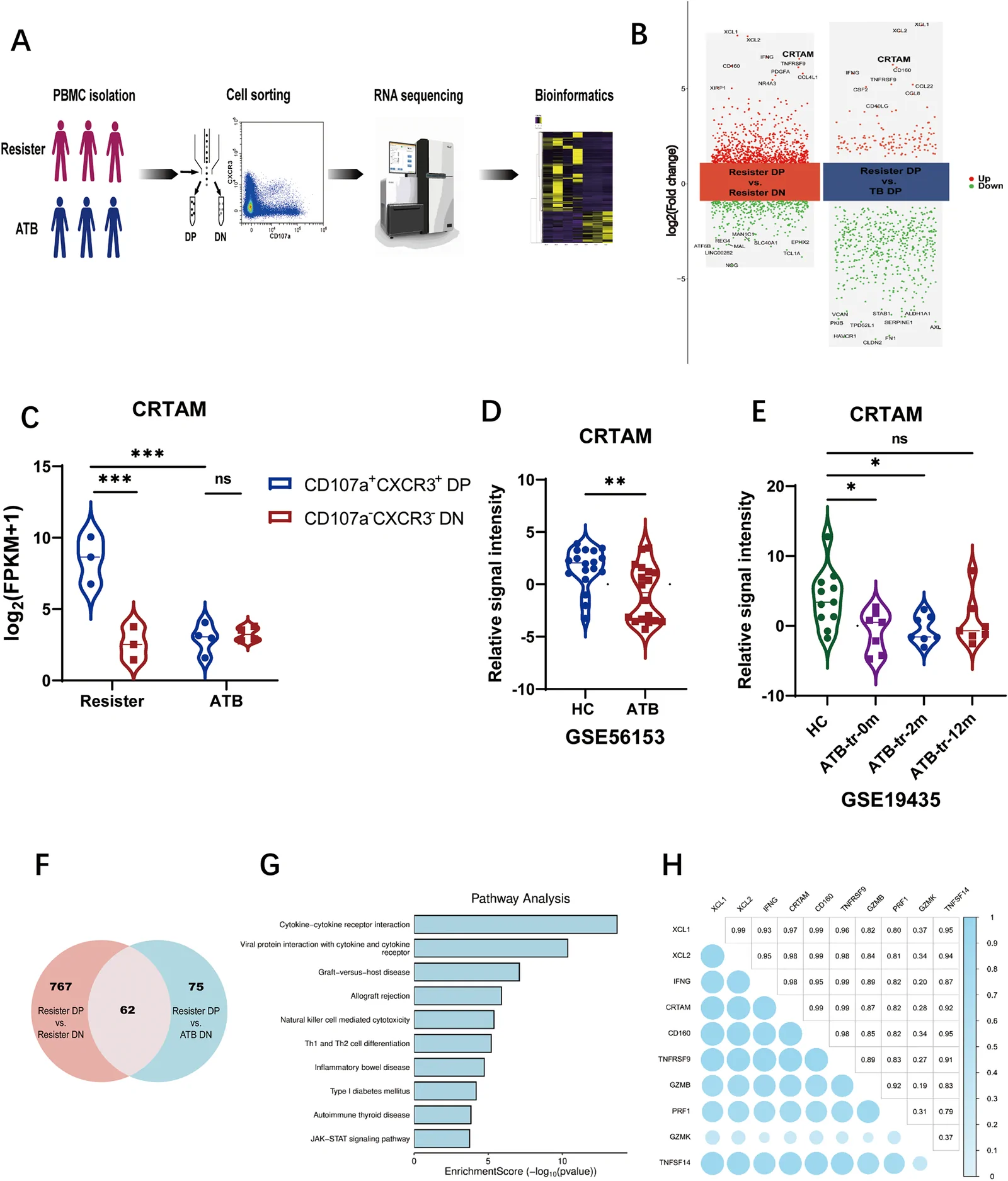
CRTAM highly expressed in lung effector lymphocytes (CXCR3^+^CD107a^+^) of Resisters and co-expressed with effector cytokines. **(A)** The schematic workflow for sample processing, cell sorting, mRNA sequencing, and bioinformatics analysis. **(B)** Differentially expressed genes between CXCR3^+^CD107a^+^ (double-positive, DP) and CXCR3^-^CD107a^-^ (double-negative, DN) cell subsets from Resisters subjects and ATB patients. **(C)** CRTAM expression levels between DP and DN cell subsets in Resisters subjects and ATB patients. **(D, E)** Expression levels of CRTAM in PBMCs were analyzed by the GEO2R platform with GSE56153 **(D)**, GSE19435 **(E)** in HC subjects, ATB patients or ATB treated for 0, 2, 12 months, respectively. **(F)** Venn diagram shows highly expressed genes in Resisters DP comparing with Resisters DN or TB DP cells subsets. **(G)** KEGG pathway analysis of 62 common highly expressed genes in Resisters DP cell subsets. **(H)** Correlation analysis of the top 10 highly expressed genes among these 62 common highly expressed genes Resisters DP cell subsets. Results are expressed as mean ± SEM. Statistical significance was determined by two-way ANOVA followed by Šídák’s multiple-comparisons test **(C)**, unpaired Student’s *t*-test **(D)**, or one-way ANOVA followed by Dunnett’s multiple-comparisons test **(E)**. ns, not significant; *p < 0.05, **p < 0.01, ***p < 0.001; HC, heathy controls; ATB, active tuberculosis.

CRTAM has been robustly implicated in immune-related processes across a range of diseases, including cancers (22–25), viral infections (26, 27), and other immune-dysfunction diseases (28–30). However, its role in the context of *Mtb* infection remains virtually unexplored. To further observe the relation of CRTAM with *Mtb* infection, we initially evaluated the expression of CRTAM in ATB patients and healthy subjects (31, 32). Results showed that the expression of CRTAM was higher in HC subjects than in ATB patients (**Figure 1D**). And the expression of CRTAM in ATB patients increased progressively during anti-TB treatment. At 12 months after treatment, the expression of CRTAM no longer significantly differed from that in HC (**Figure 1E**). The above results showed that CRTAM expression was depressed in TB infection, and would be restored to near-normal levels under anti-TB treatment. It implied that CRTAM expression was positively associated with protective immune responses against *Mtb* infection.

Furthermore, we found that the 62 upregulated genes in Resister DP cells comparing with Resister DN cells and ATB DP cells (**Figure 1F**) were mainly clustered in cell-cell interactions, NK cell-mediated cytotoxicity pathways and related processes (**Figure 1G**). Correlation analysis of the top 10 differentially expressed genes demonstrated significant positive associations between expression of CRTAM and multiple cytotoxicity-associated molecules, activation markers, including IFN-γ, granzyme B, and TNFRSF9 (**Figure 1H**). This meant that CRTAM might be closely associated with effector function of lymphocytes during mycobacterial infection.

### CRTAM rapidly expressed in activated T cells upon stimulation with *Mtb* antigens

CRTAM is expressed in a TCR-dependent manner, which serves as an early activation marker (17). To profile CRTAM expression in T cells, we stimulated HC-derived PBMCs with anti-CD3/CD28 antibodies and measured CRTAM expression as the frequencies of CRTAM^+^ cells in CD3^+^, CD4^+^ and CD8^+^ T cell populations over time. Whereas CRTAM expression in fresh naive T cells was extremely low prior to anti-CD3/CD28 antibodies stimulation, CRTAM was promptly expressed beginning at 6 h after stimulation. In CD3^+^ T cells, about 15~20% of cells expressed CRTAM (**Figure 2A**). However, less than 1% of CD4^+^ T cells expressed CRTAM regardless of stimulation (**Figure 2B**). Accordingly, more than 25~40% of CD8^+^ T cells expressed CRTAM after 6 h stimulation, with such high expression being sustainable till 24 h (**Figure 2C**). These results showed that CRTAM would rapidly express in the early stage of T cell activation induced by anti-CD3/CD28 antibodies, particularly in activated CD8^+^ T cells, which was consistent with the role in rapid early control of *Mtb* infection in Resisters.

**Figure 2:**
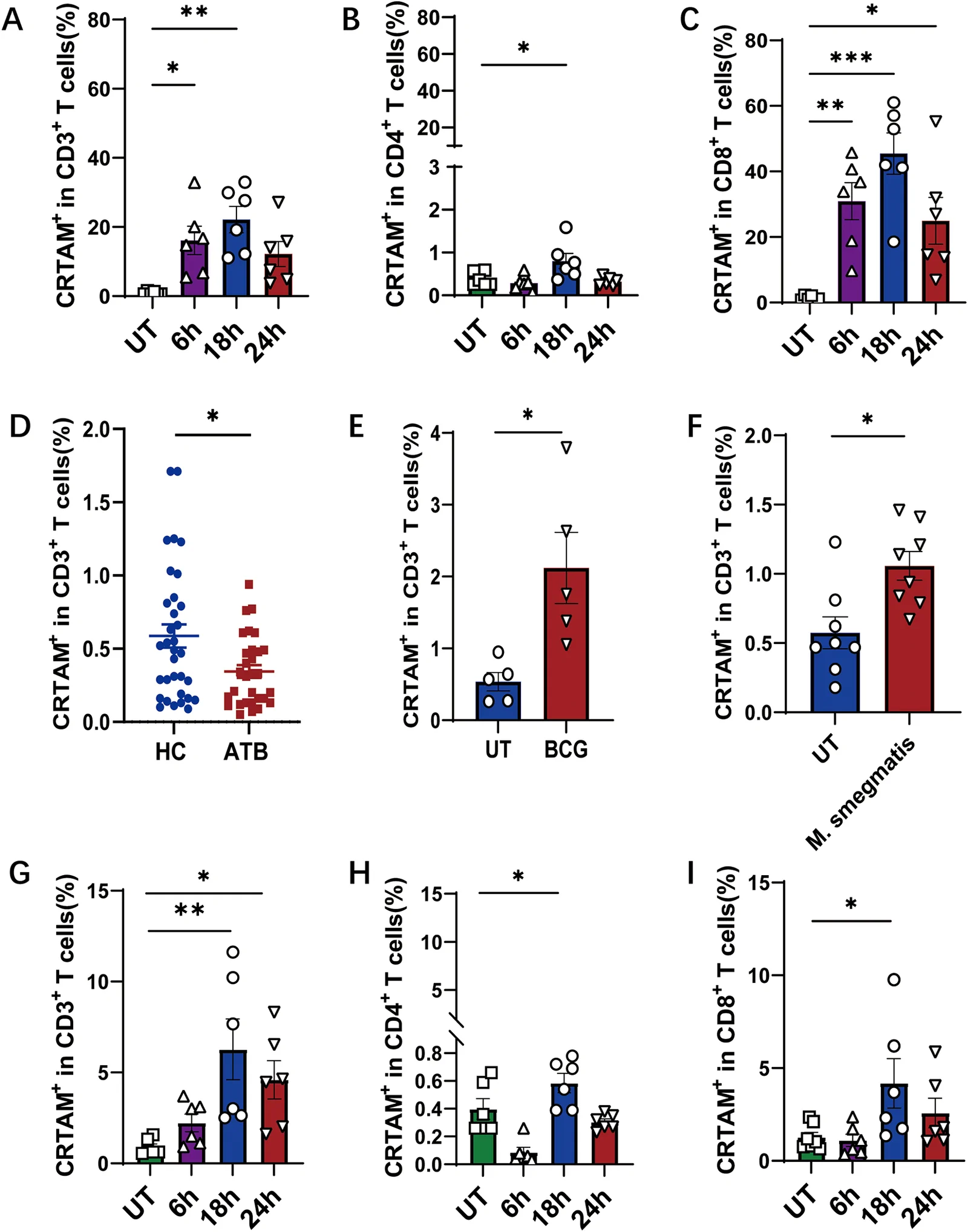
CRTAM rapidly expressed in activated T cells, especially CD8^+^ T cells, stimulated by *Mtb* antigens. **(A–C)** Frequencies of CRTAM^+^ cells in CD3^+^ **(A)**, CD4^+^ **(B)**, and CD8^+^ **(C)** T cells were determined by flow cytometry methods when HC PBMCs (n = 6) were stimulated with anti-CD3/CD28 (2 μg/mL) for the indicated hours (h). **(D)** Frequencies of CRTAM^+^ cells in CD3^+^ T cells of HC (n = 33) and ATB (n = 31) PBMCs were determined by flow cytometry methods. **(E)** Frequencies of CRTAM^+^ cells in CD3^+^ T cells were determined by flow cytometry methods when HC PBMCs (n = 5) were stimulated with BCG (MOI = 1) for 4 h. **(F)** Frequencies of CRTAM^+^ cells in CD3^+^ T cells were determined by flow cytometry methods when HC PBMCs (n = 8) were stimulated with *M. smegmatis* (MOI = 1) for 4 h. **(G–I)** Frequencies of CRTAM^+^ cells in CD3^+^ **(G)**, CD4^+^ **(H)**, and CD8^+^ **(I)** T cells were determined by flow cytometry methods when HC PBMCs (n = 6) were stimulated with H37Rv lysate (10 μg/mL) for the indicated hours (h). Results are expressed as mean ± SEM. Each symbol represents an independent biological replicate. Statistical significance was determined by one-way ANOVA followed by Dunnett’s multiple-comparisons test **(A–C, G–I)**, unpaired Student’s *t*-test **(D)**, or paired Student’s t-test **(E, F)**. *p < 0.05, **p < 0.01, ***p < 0.001; UT, untreated.

To investigate the correlation of CRTAM expression with *Mtb* infection, we assessed CRTAM expression in T cells of both HC and ATB subjects, both at baseline and following antigen stimulation. In the absence of antigen stimulation, the frequency of CRTAM^+^ cells among CD3^+^ T cells was significantly lower in ATB patients than in HC subjects (**Figure 2D**), suggesting that chronic *Mtb* infection may lead to downregulation of CRTAM. To determine whether mycobacterial antigens drive CRTAM expression, we measured frequencies of CRTAM^+^ cells in T cells of HC-derived PBMCs stimulated with BCG, *M. smegmatis*, and *Mtb* H37Rv lysate, respectively. Results showed that BCG stimulation increased the frequency of CRTAM^+^ T cells four-fold of that without stimulation (**Figure 2E**). Stimulation with *M. smegmatis* also induced high CRTAM expression (**Figure 2F**). Moreover, H37Rv lysate increased the frequency of CRTAM^+^ T cells that peaked at 18 h post-stimulation (**Figures 2G–I**), and H37Rv lysate also induced CRTAM significantly expression in CD8^+^ T cells similar to anti-CD3/CD28 antibodies stimulation, with only low levels detected in CD4^+^ T cells (**Figures 2H, I**). Subsequently, we analyzed T cell phenotypes under stimulation with either anti-CD3/CD28 antibodies or H37Rv lysate. CRTAM^+^ T cells exhibited phenotypic profiles similar to their CRTAM^-^ counterparts, both being predominantly naïve (**Supplementary Figure S1**).

These findings indicated that CRTAM was highly expressed on activated T cells induced by *Mtb* antigens. However, its expression was reduced in patients with chronic active tuberculosis, suggesting that CRTAM may have been rapidly upregulated during the early stage of infection to facilitate bacterial clearance, prolonged chronic antigenic stimulation led to the exhaustion and subsequent decline of CRTAM expression. In summary, CRTAM expression was closely associated with *Mtb* infection.

### CRTAM expression in T cells appeared to modulate anti-*Mtb* effector functions leading to intracellular *Mtb* growth inhibition in macrophages

To elucidate the mechanism by which CRTAM regulates T cells to control *Mtb* growth in macrophages, we isolated CRTAM^+^ and CRTAM^-^ CD3^+^ T cells from HC-derived PBMCs stimulated with anti-CD3/CD28 antibodies and co-cultured them with autologous H37Rv-infected hMDMs for 3 days. Bacterial burdens in CRTAM^+^ T cell co-culture groups were reduced by ~25% compared with those in CRTAM^-^ T cells groups (**Figure 3A**, left). Similarly, co-culture with CRTAM^+^ T cells decreased BCG CFUs in macrophages by ~50% compared with CRTAM^-^ T cells co-culture (**Figure 3A**, right). These results demonstrated that CRTAM^+^ T cells presented more potent cytotoxic ability than their CRTAM^-^ counterparts.

**Figure 3:**
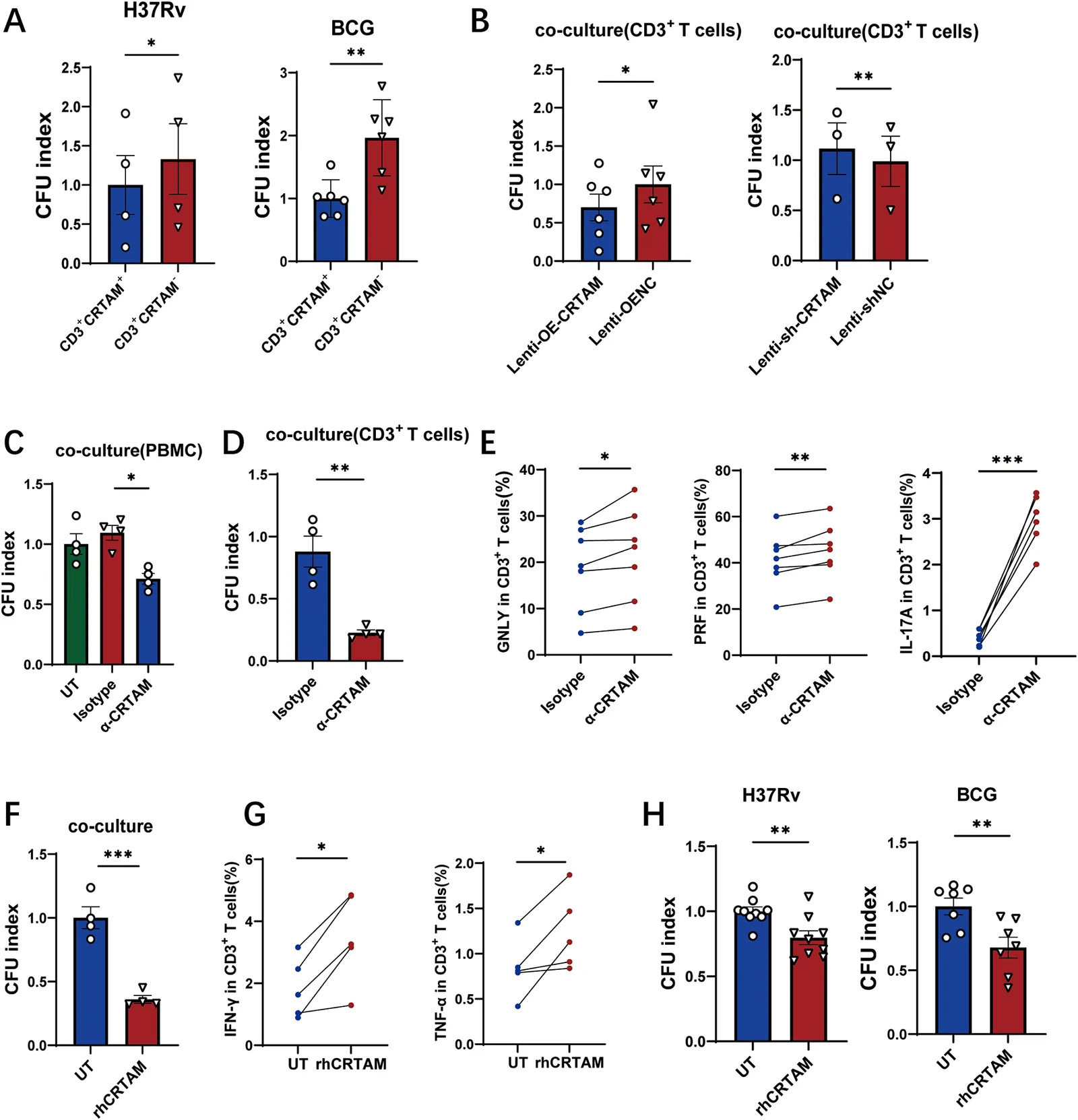
CRTAM expression in T cells modulated anti-*Mtb* functions inhibiting intracellular *Mtb* growth in macrophages. **(A)** CD3^+^CRTAM^+^ and CD3^+^CRTAM^-^ T cells isolated from HC PBMCs (n = 4) stimulated by anti-CD3/CD28 were co-cultured with H37Rv-infected hMDMs (left) and BCG-Infected THP-1 (right) for 3 days, and intracellular bacterial survival in hMDMs was quantified by CFU assay. **(B)** CD3^+^ T cells purified from HC PBMCs (n = 6) transduced with lentivirus to overexpress CRTAM (left, Lenti-OE-CRTAM) or to knockdown CRTAM expression (right, Lenti-sh-CRTAM) were co-cultured with H37Rv-infected hMDMs (MOI = 1) for 3 days, and intracellular bacterial survival in hMDMs was quantified by CFU assay. **(C, D)** HC PBMCs **(C)** or sorted CD3^+^ T cells **(D)** were co-cultured with H37Rv-infected hMDMs (MOI = 1) for 3 days in the presence of anti-CRTAM antibody (Cr24.1) or Isotype (10 μg/mL), and intracellular bacterial survival in hMDMs was quantified by CFU assay. **(E)** The frequencies of GNLY (left), PRF (middle), and IL-17A (right) producing CD3^+^ T cells from HC PBMCs (n = 7) co-cultured with BCG-infected THP-1 in the presence of anti-CRTAM antibody or Isotype (10 μg/mL) were determined by flow cytometry methods. **(F)** HC PBMCs (n = 4) were co-cultured with H37Rv-infected THP-1 in the presence of recombinant human CRTAM (rhCRTAM) protein (20 μg/mL) for 72 h, and intracellular bacterial survival in macrophages was quantified by CFU assay. **(G)** The percentages of IFN-γ (left) and TNF-α (right) producing CD3^+^ T cells in HC PBMCs (n = 5) co-cultured with BCG-infected THP-1 in the presence of rhCRTAM protein (20 μg/mL) for 24 h were determined by flow cytometry methods. **(H)** H37Rv-infected hMDMs (left) and BCG-infected THP-1 (right) were cultured in the presence of rhCRTAM protein (20 μg/mL) for 72 h, and intracellular bacterial survival in macrophages was quantified by CFU assay. Results are expressed as mean ± SEM. Each symbol represents an independent biological replicate. Statistical significance was determined by paired Student’s t-test **(A, B, D–H)** or one-way ANOVA followed by Tukey’s multiple-comparisons test **(C)**. *p < 0.05, **p < 0.01, ***p < 0.001; UT, untreated; GNLY, granulysin; PRF, perforin.

To determine the effect of CRTAM on T cells effector functions, we manipulated CRTAM expression in HC-derived CD3^+^ T cells using lentiviral vectors. CRTAM was overexpressed via Lenti-OE-CRTAM (lentivirus overexpressing *CRTAM*) and knocked down via Lenti-sh-CRTAM (lentivirus expressing CRTAM-specific shRNA). After confirming transduction efficacy by quantitative PCR (**Supplementary Figure S2A**), the altered T cells were co-cultured with H37Rv-infected hMDMs to evaluate their effects on intracellular *Mtb* growth. Results showed that overexpression of CRTAM reduced bacterial counts by approximately 30% compared with T cells transduced with Lenti-OENC (empty lentivirus as negative control, **Figure 3B**, left). Conversely, H37Rv CFU counts in co-culture with T cells transduced with Lenti-sh-CRTAM were significantly increased compared with controls (**Figure 3B**, right). Similar results were observed in co-culture systems using PBMCs with altered CRTAM expression incubated with BCG-infected THP-1 macrophages (**Supplementary Figure S2B**). These findings confirmed that CRTAM expression directly modulated the ability of T cells to inhibit intracellular mycobacterial growth in macrophages.

To investigate whether CRTAM expression modulates the ability of lymphocytes to inhibit intracellular mycobacterial growth, we co-cultured H37Rv-infected hMDMs with HC-derived PBMCs or CD3^+^ T cells stimulated with agonistic anti-CRTAM antibody (α-CRTAM). Results showed that there were about 35% reduction in H37Rv CFU counts of CRTAM agonist treatment compared with those with isotype control (**Figure 3C**). Similarly, CD3^+^ T cells treated with anti-CRTAM agonist antibody also exhibited more potent activity in inhibiting intracellular H37Rv growth than isotype-treated cells (**Figure 3D**). Concurrently, CD3^+^ T cells treated with anti-CRTAM agonist antibody showed obvious increase in production of effector molecules, including granulysin, perforin, and IL-17A (**Figure 3E**).

Furthermore, we treated THP-1 cells with exogenous recombinant human CRTAM protein and assessed its effect on mycobacterial growth. We found that BCG CFU counts in co-cultures of CD3^+^ T cells and BCG-infected THP-1 cells treated with recombinant human CRTAM were approximately 30% of those in the control group (**Figure 3F**). Production of IFN-γ and TNF-α by CD3^+^ T cells was also significantly increased following recombinant human CRTAM treatment (**Figure 3G**). Moreover, exogenous recombinant CRTAM reduced intracellular *Mtb* growth in hMDMs and intracellular BCG growth in THP-1-derived macrophages (**Figure 3H**). However, in a cell-free bacterial growth assay, recombinant CRTAM did not inhibit the growth of *Mtb* and *M. smegmatis* in the absence of macrophages and T cells (**Supplementary Figures S2C**, **S2D**). These findings argue against a direct antimycobacterial activity of recombinant CRTAM itself and suggest that recombinant CRTAM may enhance host-cell-dependent control of intracellular mycobacterial growth, although the underlying mechanism remains to be determined.

### CRTAM^+^ T cells exhibit high anti-tuberculosis cytokines production

To characterize the transcriptional profile of CRTAM^+^ T cells, we performed RNA sequencing analysis in CD3^+^CRTAM^+^ (Double Positive, DP) and CD3^+^CRTAM^-^ (Single Positive, SP) subpopulations isolated from healthy donors following overnight anti-CD3/CD28 activation (**Figure 4A**). Transcriptional analysis revealed that CD3^+^CRTAM^+^ cells exhibited elevated expression of genes associated with natural killer cell-mediated cytotoxicity, chemokine signaling, and the phosphoinositide-3-kinase (PI3K)-AKT pathway, among others (**Figures 4B–D**). These findings suggested that CRTAM^+^ T cells possessed a transcriptional signature indicative of potent cytotoxic effector function and associated with chemotactic function.

**Figure 4:**
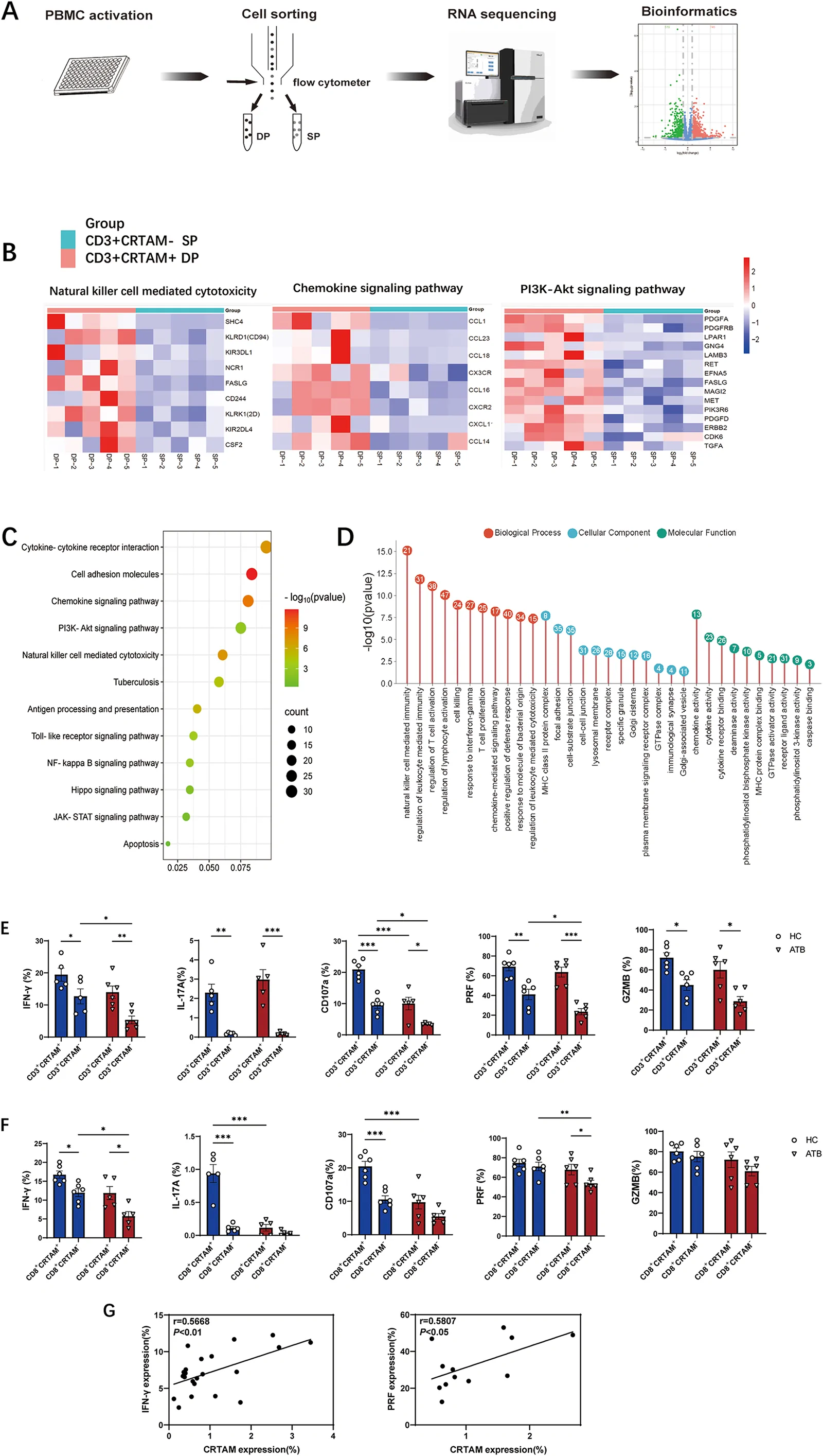
CRTAM^+^ T cells highly expressed effector molecules with anti-tuberculosis activity. **(A)** A schematic overview of the experimental workflow: PBMCs were stimulated with anti-CD3/CD28 for 18 h, after which CD3^+^CRTAM^+^ (Double-positive, DP) and CD3^+^CRTAM^-^ (Single-positive, SP) T cells were sorted for RNA sequencing. **(B)** Heatmap depicting the differential expression of genes involved in the indicated signaling pathways based on KEGG enrichment among CD3^+^CRTAM^+^ T cells (DP) and CD3^+^CRTAM^-^ T cells (SP). **(C, D)** KEGG pathway **(C)** and GO **(D)** enrichment analysis of up-regulated genes in CD3^+^CRTAM^+^ T cells, compared with CD3^+^CRTAM^-^ T cells. **(E)** Percentages of IFN-γ-, IL-17A-, CD107a-, Perforin-, and Granzyme B-producing cells in CD3^+^CRTAM^+^ T cells of HC and TB subjects, co-cultured with BCG-infected hMDMs, were determined by flow cytometry methods (n = 5–7 per group). **(F)** Percentages of IFN-γ-, IL-17A-, CD107a-, Perforin-, and Granzyme B-producing cells in CD8^+^CRTAM^+^ T cells of HC and TB subjects, co-cultured with BCG-infected hMDMs, were determined by flow cytometry methods (n = 5–7 per group). **(G)** Correlation analysis of expression levels between CRTAM and other cytokines including IFN-γ and Perforin based on FACS data. Results are expressed as mean ± SEM. Each symbol represents an independent biological replicate. Statistical significance was determined by Two-way ANOVA followed by Tukey’s multiple-comparisons test **(E, F)** or Pearson correlation analysis **(G)**. *p < 0.05, **p < 0.01, ***p < 0.001; PRF, perforin; GZMB, granzyme B.

To assess the effector functions of CRTAM^+^ T cells, we co-cultured PBMCs with BCG-infected macrophages, a model that efficiently activates T cells to produce functional molecules including IFN-γ, TNF-α, perforin, granzyme B, and granulysin (**Supplementary Figure S3**) (19, 33). We found that CRTAM^+^CD3^+^ T cells, from both PBMCs of healthy donors and ATB patients, produced more Th1-cytokine IFN-γ than their CRTAM^-^ counterparts (**Figure 4E**). Results also showed that the frequency of IL-17A-producing cells in CRTAM^+^CD3^+^ T cells was approximately 10-fold higher than that in CRTAM^-^ counterparts (**Figure 4E**). These indicated that CRTAM^+^CD3^+^ T cells presented a robust Th1/Th17 cytokine profile. Moreover, results also showed that the frequencies of CD107a-, perforin-, and granzyme B-producing cells in CRTAM^+^CD3^+^ T cells were all significantly higher than those in CRTAM^-^CD3^+^ cells, demonstrating that CRTAM^+^CD3^+^ cells produce more cytotoxic-related molecules (**Figure 4E**). Similarly, CRTAM^+^CD8^+^ T cells, like CRTAM^+^CD3^+^ T cells, also showed increased production of Th1-type, Th17-type and cytotoxic molecules (**Figure 4F**).

Furthermore, correlation analysis confirmed that CRTAM expression levels were positively correlated with both IFN-γ and perforin expression (**Figure 4G**), consistent with transcriptomic data and indicative of an enhanced capacity to control intracellular bacterial growth. Taken together, these results demonstrated that CRTAM^+^ T cells display enhanced Th1/Th17-associated cytokine responses and cytotoxic-associated molecules and are associated with improved control of intracellular mycobacterial growth.

### *CRTAM*-deficient mice were more susceptible to *Mtb* infection

The preceding experiments indicated that CRTAM was associated with enhanced control of *Mtb* infection *in vitro*. To investigate the role of CRTAM in host anti-tuberculosis immune responses *in vivo*, we challenged mice with mycobacteria and examined the expression profile of CRTAM. Following intravenous BCG infection, the expression level of CRTAM on T cells in mouse splenic lymphocytes was significantly increased about 1.6 folds, with similar results observed in CD8^+^ T cells (**Figure 5A**). Consistent results were observed in mice infected intravenously with fast-growing *M. smegmatis* (**Supplementary Figure S4A**, **S4B**).

**Figure 5:**
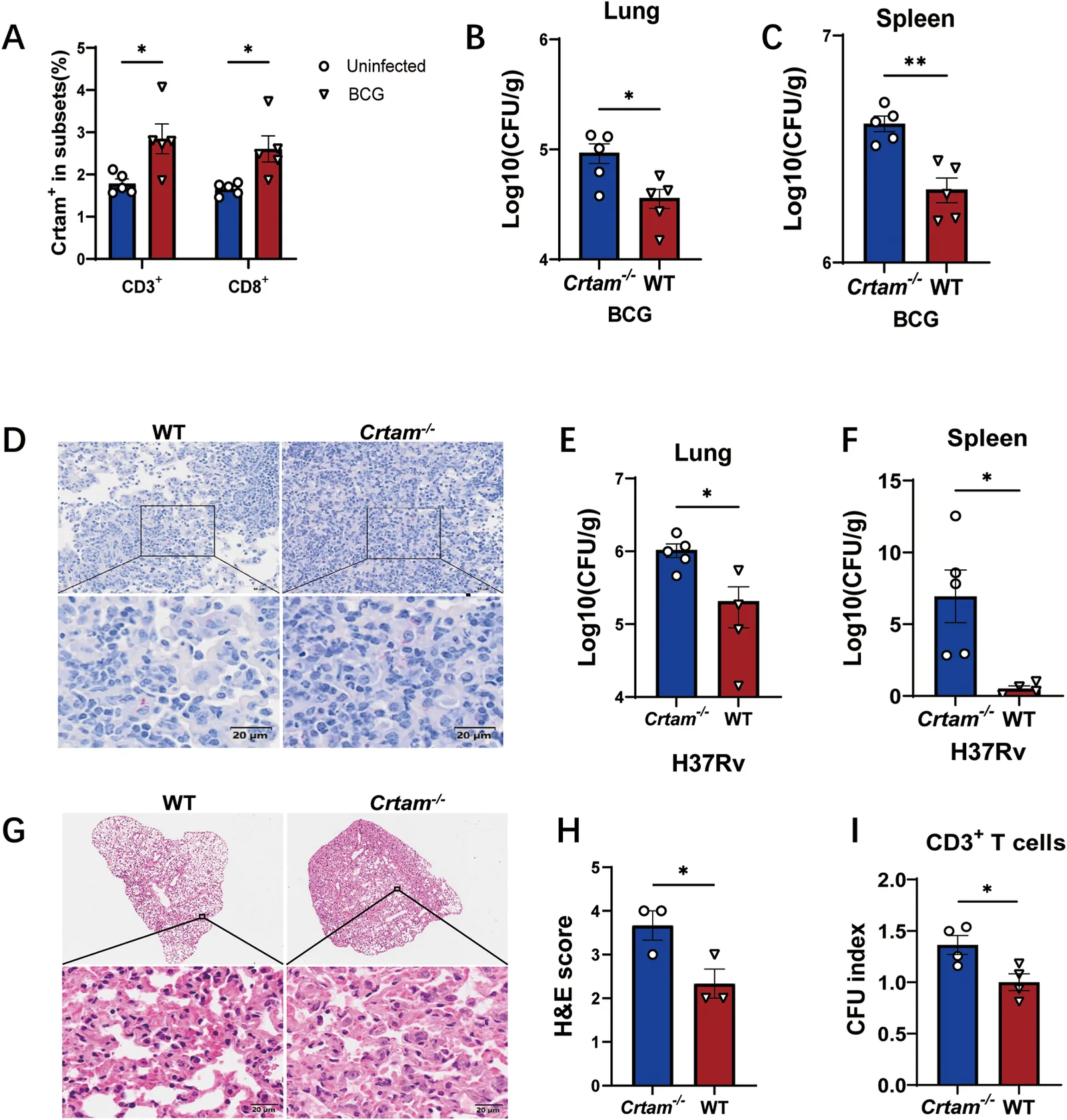
*Crtam*-deficient mice were more susceptible to *Mtb* infection. **(A)** Percentages of Crtam^+^ T cell subsets in splenic lymphocytes were analyzed by flow cytometry in WT mice following a 2-week BCG intravenous infection (1×10^6^ CFU, tail vein) or without infection (n = 5 per group). **(B, C)** Bacterial CFUs in the lungs **(B)** and spleens **(C)** of *Crtam^-/-^* and WT mice following a 4-week BCG infection (1×10^6^ CFU, i.v.) (n = 5 per group). **(D)** Histopathological analysis of lung sections from H37Rv-infected mice was performed using Ziehl-Neelsen staining (scale bars, 50 μm (upper) and 20 μm (lower)). **(E, F)** Bacterial CFUs in the lungs**(E)** and spleens **(F)** of *Crtam^-/-^* and WT mice following a 4-week *Mtb* H37Rv infection (1×10^4^ CFU, i.n.) (n = 4–5 per group). **(G)** Histopathological analysis of lung sections from H37Rv-infected mice was performed using H&E staining (scale bars, 2 mm (upper) and 20 μm (lower)). **(H)** Histological scores of lung sections (n = 3 per group). **I:** *Mtb* survival in RAW264.7 macrophages co-cultured with CD3^+^ T cells sorted from *Crtam*^-/-^ and WT mice for 72 h (n = 4 per group). Results are expressed as mean ± SEM. Each symbol represents an independent biological replicate. Statistical significance was determined by Two-way ANOVA followed by Bonferroni’s multiple comparisons test **(A)**, or unpaired Student’s t-test **(B, C, E, F, H, I)**. *p < 0.05, **p < 0.01; WT, wild-type; i.v., intravenously; i.n., intranasally.

To determine whether CRTAM contributes to host protection against mycobacterial infection, we next compared *Crtam*-deficient (*Crtam^-/-^*) mice with wild-type (WT) mice. Following intravenous BCG infection, *Crtam^-/-^* mice exhibited significantly higher bacterial burdens in both the lungs and spleens at 4 weeks post-infection compared with WT mice (**Figures 5B, C**). A similar increase in bacterial burden was observed in *Crtam^-/-^* mice following intravenous infection with fast-growing *M. smegmatis* (**Supplementary Figure S4C**, **S4D**).

We next investigated the contribution of CRTAM to pulmonary host defense against virulent *Mtb*. Mice were infected intranasally with *Mtb*, and bacterial burdens and pulmonary pathology were assessed at 4 weeks post-infection. *Crtam^-/-^* mice exhibited significantly increased bacterial CFU in both the lungs and spleens (**Figures 5D–F**) and more severe lung inflammatory damage (**Figures 5G, H**) compared with infected wild-type mice. Together, these findings indicate that CRTAM deficiency is associated with increased susceptibility to mycobacterial infection and impaired pulmonary control of *Mtb*.

To further examine whether CRTAM expression in T cells contributes to their antimycobacterial effector function, we isolated CD3^+^ T cells from splenic lymphocytes of *Crtam^-/-^* and wild-type mice, and co-cultured them with *Mtb*-infected RAW264.7 macrophages for 3 days at an effector-to-target ratio of 10:1 *in vitro*. The results showed that the bacterial burden in the group treated with *Crtam^-/-^* CD3^+^ T cells was approximately 1.3-folds higher than that in the group treated with wild-type CD3^+^ T cells (**Figure 5I**), implicating that CRTAM contributes to T cell-mediated control of mycobacterial growth in macrophages.

### Exogenous CRTAM administration confers host protective immunity

The above results proved that CRTAM was one important protective component in host anti-mycobacterial infection, and that recombinant CRTAM protein efficiently enhanced T cell and macrophage function to inhibit intracellular *Mtb* growth *in vitro*. To further identify the protective role of CRTAM protein *in vivo*, mice were treated with recombinant mouse CRTAM (rmCRTAM) protein prior to *Mtb* infection, followed by five additional injections after infection (**Figure 6A**). rmCRTAM protein administration significantly reduced *Mtb* loads in the spleens (**Figure 6B**) and alleviated lung pathological damage (**Figures 6C, D**) compared with control mice. Thus, these results demonstrated that recombinant mouse CRTAM protein could confer immune protection against *Mtb* infection.

**Figure 6:**
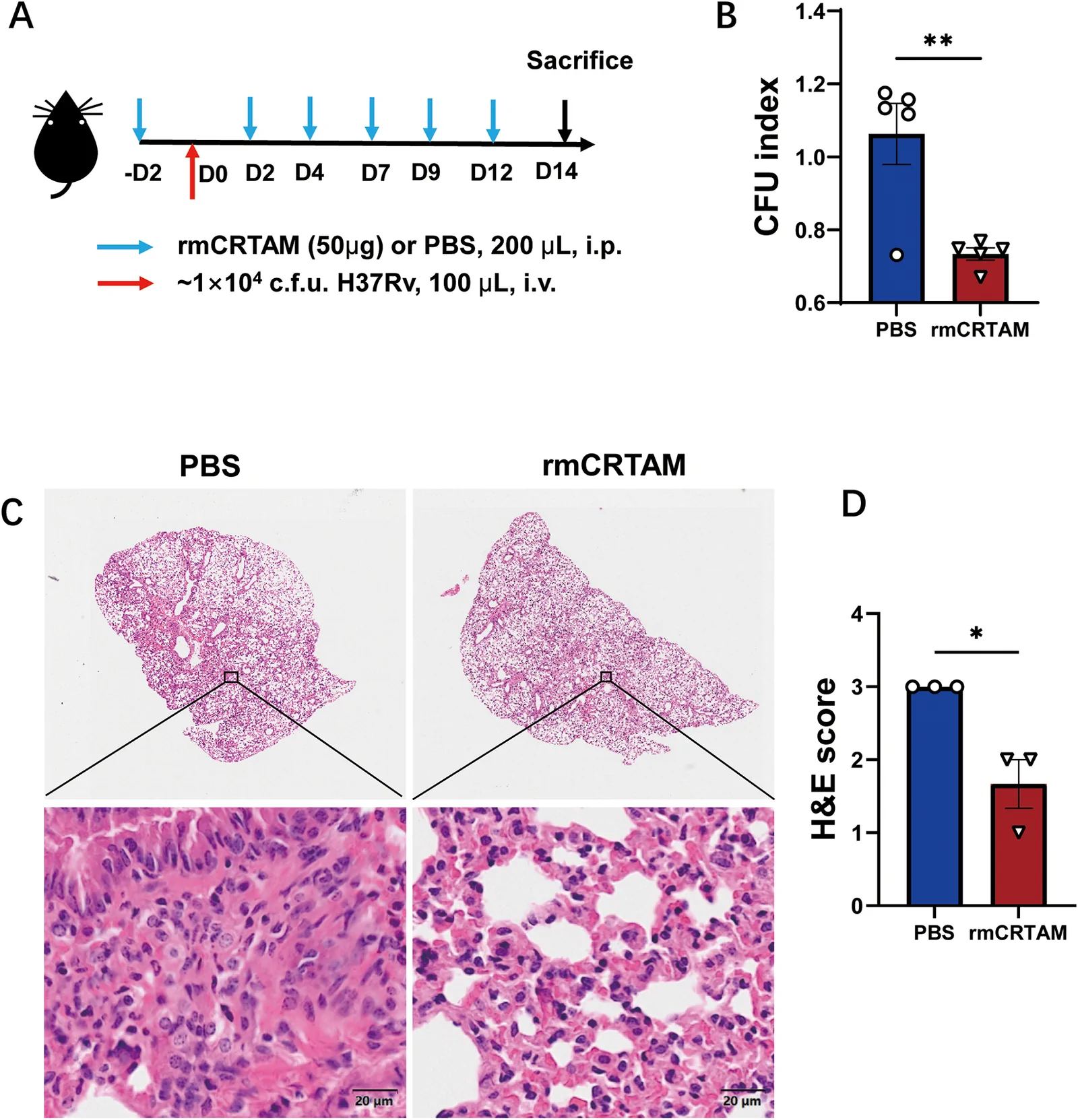
Recombinant CRTAM protein could confer immune control of *Mtb* infection in mice. **(A)** Schematic of the experimental treatment: Mice received 6 intraperitoneal injections of recombinant mouse CRTAM (rmCRTAM) protein (50 µg/mouse in 200 µL) or PBS every 2 days after H37Rv challenge (1 × 10^4^ CFU, i.v.). The first dose was administered two days prior to H37Rv challenge. **(B)** Bacterial CFUs in the spleens when WT mice were treated according to **(A)** (n = 5 per group). **(C)** Representative histopathology of inflammatory areas in the H&E-stained lung sections when WT mice were treated according to **(A)**. Scale bars, 2mm (upper), 20 μm (lower). **(D)** Histological scores of lung sections (n = 3 per group). Results are expressed as mean ± SEM. Each symbol represents an independent biological replicate. Statistical significance was determined by unpaired Student’s t-test **(B, D)**. *p < 0.05; i.v., intravenously; i.p., intraperitoneally.

## Discussion

Resisters have been proposed to represent individuals who can effectively control or clear *Mtb* following substantial exposure, making them a key population for identifying protective host immune responses against tuberculosis (7). In the present study, Resisters were defined as individuals with prolonged close contact with patients with pulmonary tuberculosis who remained negative on repeated IGRA testing. Although this phenotype is consistent with effective control or early clearance of *Mtb*, bacterial infection and subsequent clearance were not directly demonstrated in our study. To identify immune components associated with effective anti-tuberculosis responses, we administered *Mtb* PPD for *in vivo* stimulation of Resisters. From peripheral blood, we isolated CXCR3^+^CD107a^+^ double-positive (DP) cells which express markers associated with chemotactic function and cytotoxicity, for transcriptome sequencing. We found that CRTAM expression was significantly higher in DP cells than in double-negative (DN) cells, and its expression correlated strongly with effector molecules like IFN-γ, granzyme B, and perforin. These findings indicate that CRTAM and its related functional association might be closely related with host protective immune responses against *Mtb* infection.

CRTAM is primarily expressed on the surface of activated cells and is associated with cellular activation (34–36). Studies have revealed that CRTAM-expressing CD4^+^ T cells exhibit characteristics typically associated with CD8^+^ T cells and may represent precursors of CD4^+^ cytotoxic T lymphocytes (CTLs) (37). These cells are capable of secreting IFN-γ and expressing CTL-related factors such as EOMES, granzyme B, and perforin (38). CRTAM is also involved in regulating T cell polarization and can promote the infiltration of CD8^+^ T cells into tumor tissues to participate in anti-tumor immunity (22, 39). During tuberculosis infection, expression of CRTAM on NK cells was altered (40). However, the role of CRTAM on T cells during tuberculosis infection remains unclear. Based on our transcriptional profiles, we hypothesized that CRTAM-positive cells might possess enhanced anti-tuberculosis effector functions.

Consistent with previous studies (17), upon activation with anti-CD3/CD28 antibodies, CRTAM expression levels began to increase significantly as early as 6 h post-stimulation. Furthermore, we found that CRTAM expression did not change markedly in CD4^+^ T cells, while it increased significantly in activated CD8^+^ T cells. These results suggested that CRTAM expression was closely associated with T cell activation, particularly that of CD8^+^ T cells. We subsequently investigated whether the CRTAM molecule was associated with the anti-tuberculosis effector function of T cells. Under *Mtb* antigens or *Mtb* lysates for stimulation, CRTAM expression levels were increased significantly in *Mtb* antigen- activated T cells, mirroring the trend seen with anti-CD3/CD28 stimulation, and again this increase was prominent in CD8^+^ T cells. These findings indicate that CRTAM expression is closely associated with T cell activation. Importantly, this association raises the possibility that CRTAM^+^ cells may represent a more highly activated effector population rather than a population defined exclusively by CRTAM-dependent activity.

To further characterize the transcriptional features associated with CRTAM expression, we sorted CRTAM^+^ and CRTAM^-^ T cells for RNA sequencing. CRTAM^+^ T cells displayed an NK cell-like transcriptional profile enriched in cytokine and chemokine genes. Given the rapid induction of CRTAM following T-cell activation, the transcriptional differences between CRTAM^+^ and CRTAM^-^ cells may partly reflect differences in their activation and effector states. Thus, the RNA-seq analysis should be interpreted as identifying transcriptional features associated with CRTAM expression rather than establishing CRTAM as the causal driver of these transcriptional differences. Nevertheless, our functional experiments provide evidence that extends beyond this association. Purified CRTAM^+^ T cells exhibited greater anti-mycobacterial activity than CRTAM^-^ T cells, and modulation of CRTAM expression by lentiviral overexpression or knockdown altered the ability of T cells to control intracellular mycobacterial growth. Furthermore, agonistic stimulation of CRTAM enhanced the production of effector molecules and improved control of intracellular *Mtb* growth. Together, these findings suggest that CRTAM is associated with an activated effector T cell phenotype and may also contribute functionally to T cell anti-mycobacterial responses, although the extent to which CRTAM itself drives the broader transcriptional program of activated effector cells remains to be determined.

As an adhesion molecule (41), CRTAM regulates multiple immune processes including NK cell cytotoxicity, Th17 and CTL responses by engaging in intercellular adhesion with its ligand CADM1 and related partners (39, 42–45). Using a co-culture model of *Mtb*-infected macrophages and PBMCs, we found that infected macrophages likely undergo further activation and enhanced antigen-presenting capacity, which markedly increased the production of Th1-type cytokines such as IFN-γ and cytotoxic molecules by PBMCs. Within this system, CRTAM^+^ T cells, especially CD8^+^ T cells produced significantly more IFN-γ, IL-17A, CD107a, and perforin than their CRTAM^-^ counterparts. Moreover, CRTAM^+^ T cells from healthy donors generated more effector molecules than those from ATB patients. These findings suggest that CRTAM is closely associated with the effector function of *Mtb* antigen-induced T cells and that this function is significantly impaired under conditions of chronic *Mtb* infection. Although gain- and loss-of-function data support a functional contribution of CRTAM, the specific CRTAM-CADM1 interaction and downstream signaling pathway were not directly established, mechanistic studies mapping is ongoing.

Furthermore, we found that *Crtam*-deficient mice exhibit increased susceptibility to infection with both slow- and fast-growing mycobacteria, demonstrating that loss of CRTAM enhances host susceptibility to mycobacterial infection. Interestingly, treatment with recombinant CRTAM protein significantly enhanced the ability of T cells and macrophages to inhibit intracellular mycobacterial growth, and decreased *Mtb* burdens in mice while alleviating pathological damages. Recombinant CRTAM was administered before infection in the *in vivo* study, therefore, therapeutic efficacy against established TB and long-term safety, including the balance between bacterial control and immunopathology, require further investigation.

Nevertheless, several limitations of the present study should be considered. First, although the use of primary human cells from independent donor cohorts allowed us to examine donor-level responses, the number of donors in some experiments was relatively limited, larger studies involving more diverse donor populations will be needed to validate these findings. Second, although our gain- and loss-of-function experiments support a functional contribution of CRTAM to anti-mycobacterial T cell responses, the precise cellular mechanisms by which CRTAM regulates these responses remain incompletely defined. In particular, how CRTAM-mediated interactions influence T cell effector function and communication with infected macrophages *in vivo* requires further investigation. Third, the protective effects of recombinant CRTAM observed in mice provide proof-of-concept for the potential therapeutic relevance of CRTAM, whether CRTAM can improve bacterial control in established tuberculosis, and whether prolonged CRTAM activation may cause excessive inflammation or tissue damage, remains to be determined. Future studies incorporating larger longitudinal human cohorts, more detailed mechanistic analyses, and therapeutic intervention in established infection will be important to further define the predictive, mechanistic, and translational significance of CRTAM in tuberculosis.

## Conclusion

In summary, we identify CRTAM as a molecule associated with protective anti-mycobacterial effector responses, particularly in activated CD8^+^ T cells. CRTAM expression and stimulation were associated with enhanced effector molecule production and improved control of intracellular mycobacterial growth, while *Crtam* deficiency increased susceptibility to infection in mice. Furthermore, we provide an initial *in vivo* proof-of-concept evidence that exogenous recombinant CRTAM can influence infection-associated outcomes during *Mtb* infection. These findings support CRTAM as a candidate immune-regulatory molecule for further investigation, while the underlying signaling mechanism and its potential therapeutic efficacy against established TB remain to be determined.

## Data Availability

Two RNA-seq datasets were analyzed in this study. The first dataset included CXCR3^+^CD107a^+^ double-positive (DP) cells and CXCR3^+^CD107a^-^ single-positive (SP) cells from 3 Resister individuals and 4 individuals with ATB, The RNA-seq data are provided in **Supplementary Tables S1**. The second dataset included CD3^+^CRTAM^+^ and CD3^+^CRTAM^-^ T cells from 5 independent donors, with both populations obtained from each donor, The raw sequencing data are deposited in the National Omics Data Encyclopedia (NODE) database [OEP00007265].
