# Supplementary material for: CRTAM enhances T cell antimicrobial activity, particularly CD8+ T cells, against Mycobacterium tuberculosis infection
Source: Front Immunol. 2026 Oct 23;17:1964260. doi: 10.3389/fimmu.2026.1964260 (PMC13739569; doi:10.3389/fimmu.2026.1964260)
Supplement: Supplementary file 1 [file DataSheet1.pdf]

Supplementary Figure legends

Fig. S1 A-D: Phenotypes of $CRTAM^+$ and $CRTAM^-$ cells in $CD3^+$ and $CD8^+$ T cells were determined by flow cytometry methods when HC PBMCs (n = 7) were stimulated with anti-CD3/CD28 (2 μg/mL) (A-B) or H37Rv lysate (10 μg/mL) (C-D) for 18 h, respectively. Results are expressed as mean ± SEM. Each symbol represents an independent biological replicate. Statistical significance was determined by Two-way ANOVA followed by Šídák's multiple comparisons test. $^*p < 0.05$; TEMRA, Terminally Differentiated Effector Memory T cells; TEM, Effector Memory T cells; TCM, Central Memory T cells

Fig. S2 A: Quantitative PCR analysis of CRTAM expression in sorted $CD3^+$ T cells transduced with CRTAM-overexpressing (left, Lenti-OE-CRTAM) or CRTAM-knockdown (right, Lenti-sh-CRTAM) lentivirus; **B:** HC PBMCs with overexpression (left, Lenti-OE-CRTAM) or knockdown (right, Lenti-sh-CRTAM) lentivirus of CRTAM were co-cultured with BCG-infected THP-1 for 3 days, and intracellular bacterial survival in macrophages was quantified by CFU assay. **C:** *M.smegmatis* was cultured in the presence of rhCRTAM protein (20 μg/mL) for overnight, and bacterial survival was quantified by CFU assay. **D:** *Mtb* was cultured in the presence of rhCRTAM protein (20 μg/mL) for 3 days, and bacterial survival was quantified by CFU assay. Results are expressed as mean ± SEM. Each symbol represents an independent biological replicate. Statistical significance was determined by unpaired Student's t-test. $^*p < 0.05$

Fig. S3 A co-culture system containing PBMCs and BCG-infected hMDMs, which exhibited higher frequency of T cells expressing functional cytokines, including IFN-γ, TNF-α, perforin, granzyme B and granulysin, than PBMCs cultured without BCG-infected hMDMs. Results are expressed as mean ± SEM. Each symbol represents an independent biological replicate. Statistical significance was determined by Two-way ANOVA followed by Šídák's multiple comparisons test. $^*p < 0.05$, $^{**}p < 0.01$; PRF, perforin; GZMB, granzyme B; GNLY, granulysin

Fig. S4 A-B: Percentages of $Crtam^+$ and $CD69^+$ cells in $CD3^+$ T cells (A), and $Crtam^+$ cells in $CD8^+$ T cells (B) of lung lymphocytes were determined by flow cytometry methods when WT mice were intravenously infected with 1×10^6 CFU *M.smegmatis* for 3 days (n = 4 per group). **C:** Representative

histopathology of inflammatory areas in the H&E-stained lung sections when WT mice were intranasally infected with 1× 10^4 CFU H37Rv for 4 weeks or not. Scale bars,1mm, 20 μm; black arrows, the areas of inflammatory infiltrate. **D:** Bacterial CFUs in the lungs of *Crtam*$^{-/-}$ and WT mice at 3 days after tail-vein infection with *M.smegmatis* in 1× 10^6 CFU (n = 6 per group). Results are expressed as mean ± SEM. Each symbol represents an independent biological replicate. Statistical significance was determined by Two-way ANOVA followed by Bonferroni's multiple comparisons test (A) or unpaired Student's t-test (B, D). *$p < 0.05$, **$p < 0.01$, ***$p < 0.001$

Figure S1

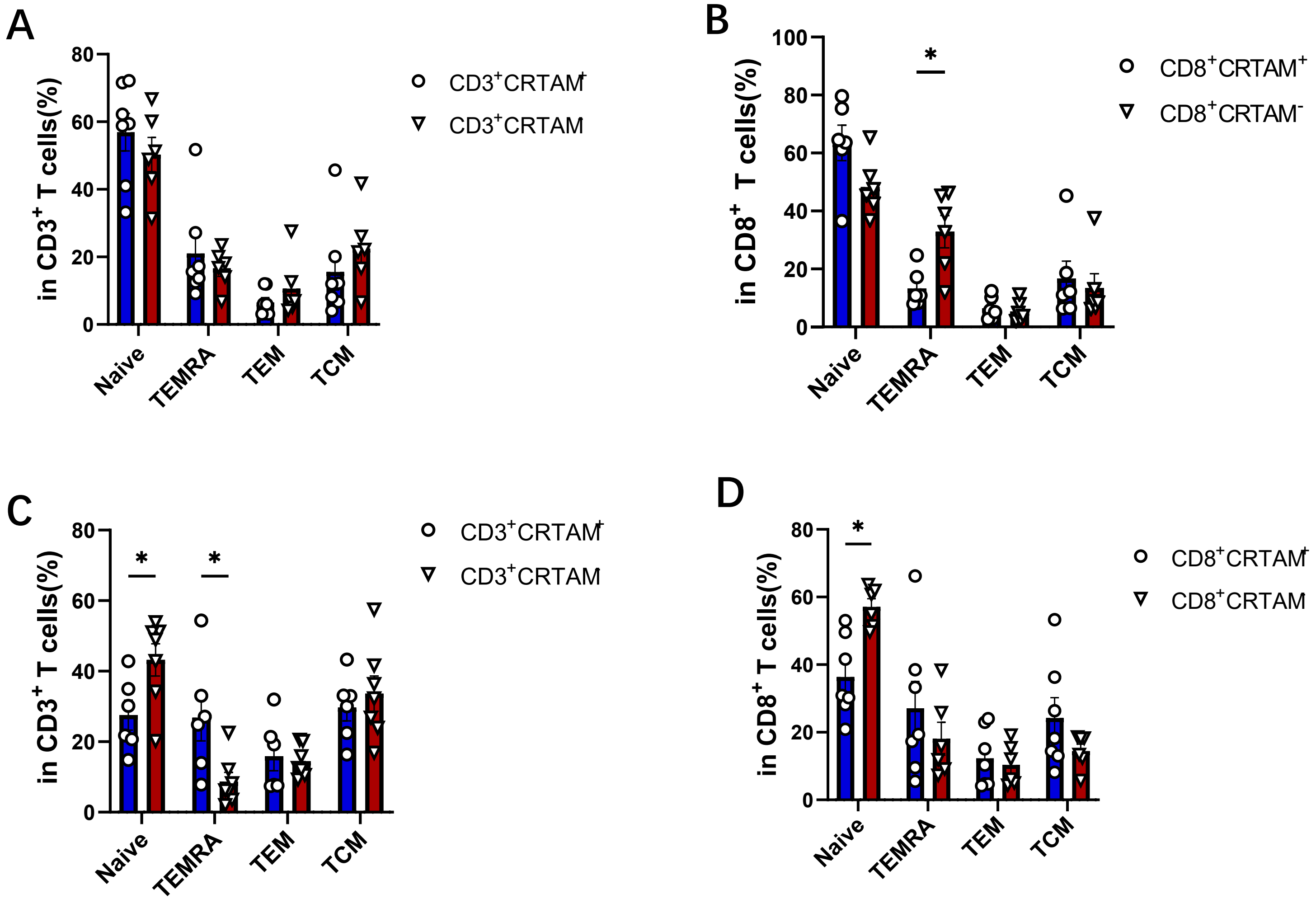

A
B
C
D
in CD3+ T cells(%)
in CD8+ T cells(%)
Naive
TEMRA
TEM
TCM
CD3+CRTAM+
CD3+CRTAM-
CD8+CRTAM+
CD8+CRTAM-

Figure S2

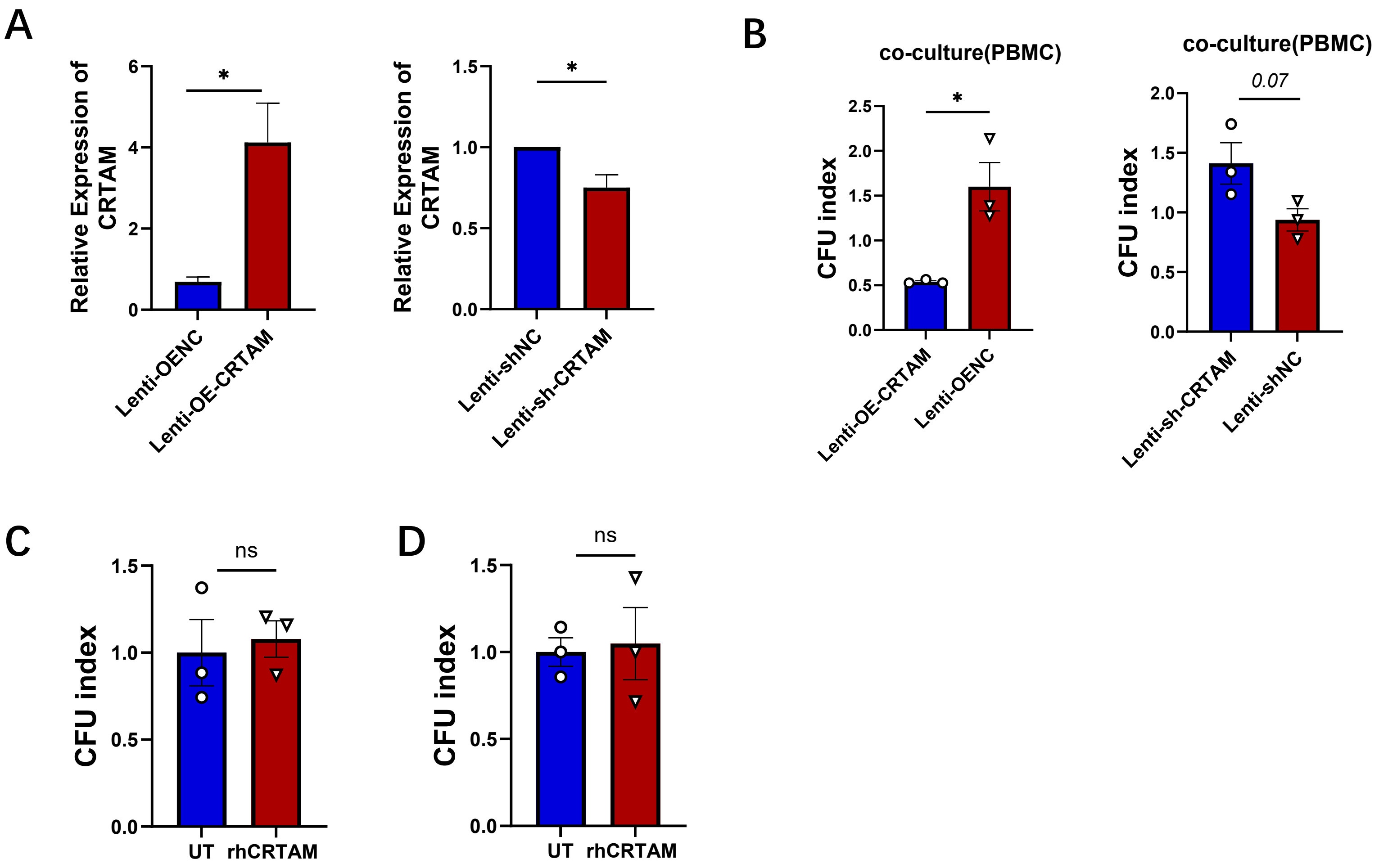
A
Relative Expression of CRTAM
6
4
2
0
*
Lenti-OENC
Lenti-OE-CRTAM
Relative Expression of CRTAM
1.5
1.0
0.5
0.0
*
Lenti-shNC
Lenti-sh-CRTAM
B
co-culture(PBMC)
CFU index
2.5
2.0
1.5
1.0
0.5
0.0
*
Lenti-OE-CRTAM
Lenti-OENC
co-culture(PBMC)
CFU index
2.0
1.5
1.0
0.5
0.0
0.07
Lenti-sh-CRTAM
Lenti-shNC
C
CFU index
1.5
1.0
0.5
0.0
ns
UT
rhCRTAM
D
CFU index
1.5
1.0
0.5
0.0
ns
UT
rhCRTAM

Figure S3

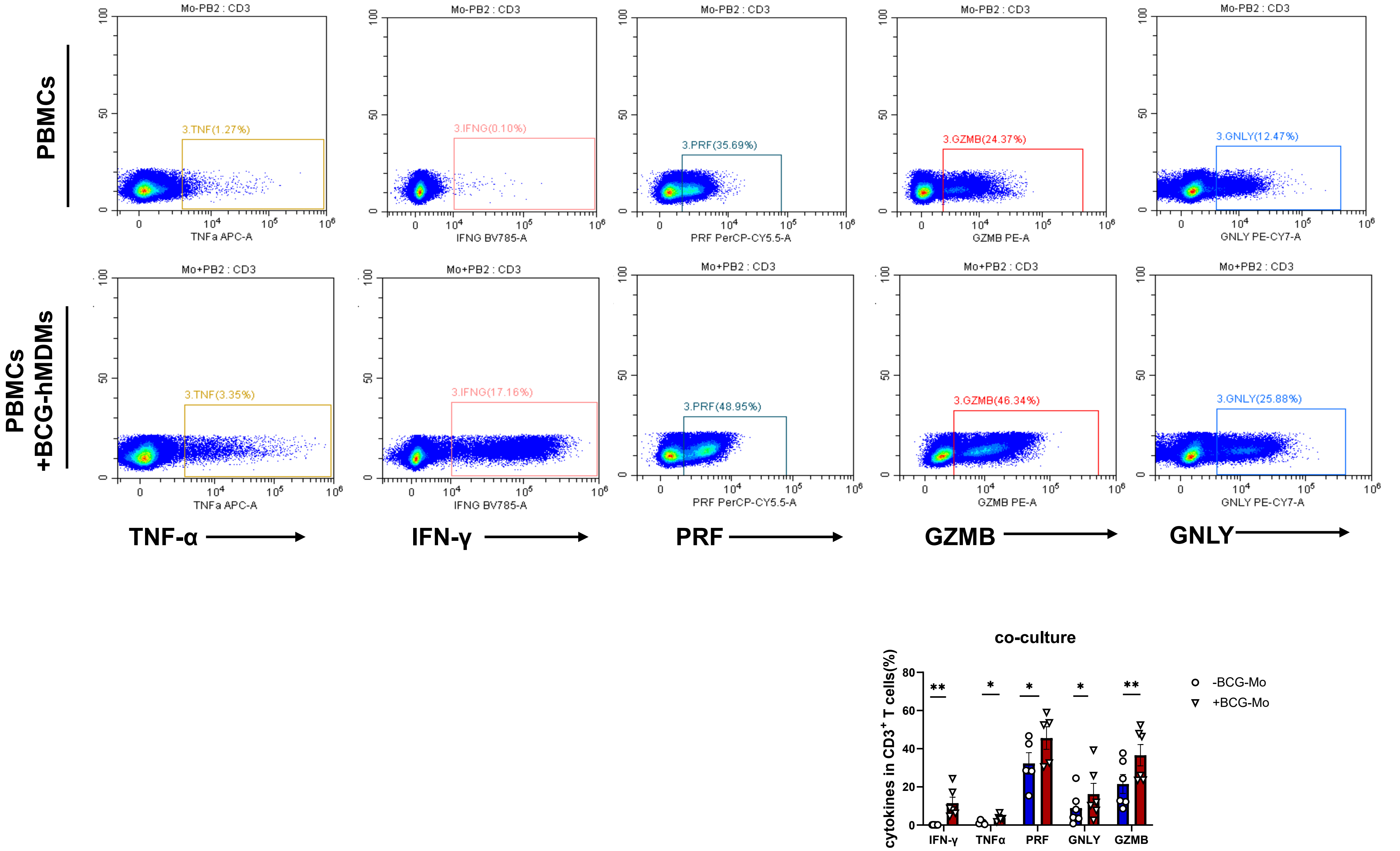
PBMCs
PBMCs +BCG-hMDMs
Mo-PB2 : CD3
Mo+PB2 : CD3
3.TNF(1.27%)
3.IFNG(0.10%)
3.PRF(35.69%)
3.GZMB(24.37%)
3.GNLY(12.47%)
3.TNF(3.35%)
3.IFNG(17.16%)
3.PRF(48.95%)
3.GZMB(46.34%)
3.GNLY(25.88%)
TNFa APC-A
IFNG BV785-A
PRF PerCP-CY5.5-A
GZMB PE-A
GNLY PE-CY7-A
TNF-α
IFN-γ
PRF
GZMB
GNLY
co-culture
cytokines in CD3+ T cells(%)
-BCG-Mo
+BCG-Mo
IFN-γ
TNFα
PRF
GNLY
GZMB

Figure S4

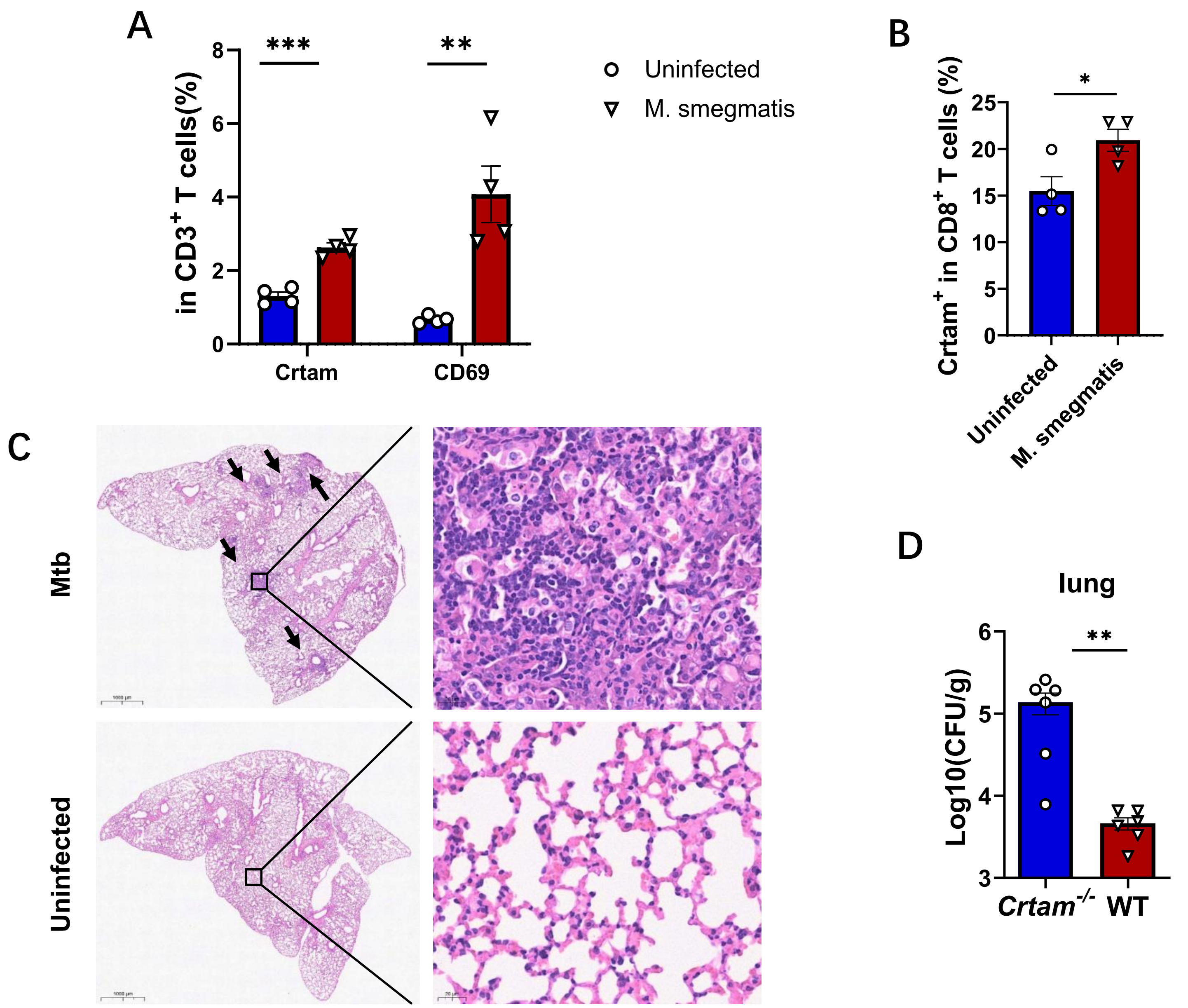
A
***
**
Uninfected
M. smegmatis
in CD3+ T cells(%)
0
2
4
6
8
Crtam
CD69
B
*
Crtam+ in CD8+ T cells (%)
0
5
10
15
20
25
Uninfected
M. smegmatis
C
Mtb
Uninfected
D
lung
**
Log10(CFU/g)
3
4
5
6
Crtam-/-
WT
